# Editorial: Medicinal drugs, the effect on memories and beyond

**DOI:** 10.3389/fphar.2024.1471049

**Published:** 2024-08-20

**Authors:** Mónica Moreira-Rodrigues, Estibaliz Ampuero, Ursula Wyneken

**Affiliations:** ^1^ Department of Immuno-Physiology and Pharmacology, Laboratory of General Physiology and Center for Drug Discovery and Innovative Medicines (MedInUP), School of Medicine and Biomedical Sciences (ICBAS), University of Porto (UP), Porto, Portugal; ^2^ Laboratory of Neurobiology of Behavior, Department of Biology, Universidad de Santiago de Chile, Santiago, Chile; ^3^ Laboratory of Neuroscience, Faculty of Medicine, Universidad de Los Andes, Santiago, Chile; ^4^ IMPACT, Center for Interventional Medicine for Precision and Advanced Cellular Therapy, Faculty of Medicine, Universidad de Los Andes, Santiago, Chile

**Keywords:** medicines, drugs, medicinal drugs, cognitive functions, memory, adverse drug reactions, psychiatry, psychology

In the fields of psychiatry, psychology, and neuroscience, cognition has a central role and is fundamental to intellectual functions. Cognitive functions include many varied and complex brain activities, and the main domains are attention, memory, and executive functions. Attention, learning, and memory are highly integrated and are dynamically interactive processes. For example, disruption of attentional abilities is reflected in impaired learning and memory. Executive functions in turn include reasoning, planning, working memory, problem-solving, and processing speed, to name just a few. This Research Topic aimed at gathering knowledge of the effects of medicinal drugs on cognitive functions in psychiatric and neurological diseases in which the use of medications can trigger desired or undesired cognitive consequences.

Medications helpful in treating infections, cancer, endocrine function, and psychiatric conditions, among other neurological diseases, have been reported to have adverse drug reactions and, thus, side effects, mimicking a state of cognitive impairment. In particular, Doxorubicin (DOX), an anticancer drug, causes cognitive impairment in cancer survivors. Alhowail et al. showed that DOX induces cognitive impairment via neuronal toxicity by upregulating α-amino-3-hydroxyl-5-methyl-4-isoxazole-propionate receptors (AMPAR) and N-methyl-d-aspartate receptors (NMDAR) expression, increasing neuroinflammation, oxidative stress, and apoptosis.

Although selective serotonin reuptake inhibitors are the only drugs approved by the Food and Drug Administration (FDA) and European Medicines Agency (EMA) to treat post-traumatic stress disorder (PTSD), it has minimal impact in decreasing PTSD symptoms and has severe side effects, hence alternative options should be explored. Martinho et al. showed that sotalol (a peripheral β-adrenoceptor antagonist) reduces traumatic memories and anxiety-like behavior in PTSD, likely due to its reduction of peripheral adrenergic activity. Sotalol may disrupt the retrieval, expression, and/or reconsolidation of contextual traumatic memory, resulting in a long-term reduction in PTSD symptoms. Furthermore, the decreased nuclear receptor Nr4a1 mRNA expression in the hippocampal formation, a transcription factor that promotes synaptic plasticity in the hippocampus, may be key to the reduced traumatic contextual memories seen after sotalol treatment.

Next, Wu et al. examined whether probucol, a drug which has antioxidant and cholesterol-lowering properties, may be beneficial in obesity-induced cognitive decline, a condition that has been consistently associated with brain insulin resistance, inflammation, and oxidative stress. Interestingly, while probucol did not alleviate high-fat diet (HFD)-induced systemic metabolic parameters, systemic oxidative stress markers, or depressive-like behaviors in mice, cognitive abilities (i.e., spatial learning and memory and social behaviors) improved in probucol-treated HFD-fed mice. In the brain, probucol decreased iNOS (a marker of neuroinflammation) in the cerebral cortex while restoring the phosphorylation level of AKT in the hippocampus. This study underscores the complexity of the relationship between obesity and brain health, highlighting the significance of preserving cognitive functions and overall brain health in affected individuals using novel therapeutic interventions.

Alzheimer’s Disease (AD) is a neurodegenerative disorder characterized by progressive memory loss and cognitive decline. The current treatment landscape for AD includes acetylcholinesterase inhibitors, NMDA receptor antagonists, monoclonal antibody therapies, immunotherapies, and anti-inflammatory strategies. However, better or alternative treatments are urgently needed. Kong et al. conducted a systematic review and meta-analysis to assess the effects of Glucagon-like peptide 1 agonists (i.e., agonists at GLP-1 receptors), which are used for the treatment of type 2 diabetes, in AD animal models. The results provide important insights into the therapeutic potential of GLP-1 receptor agonists, which exert neuroprotective effects, ameliorating disease progression and reducing severity. Factors such as gender, route of administration (e.g., intranasal), and potential mechanisms of action are comprehensively discussed.

Fluoxetine, a widely used antidepressant and selective serotonin reuptake inhibitor, has demonstrated high efficacy but has several impacts on cognition. Ampuero et al. reviewed the complex effects of fluoxetine on learning and memory, attention, and executive function. Fluoxetine’s effects on cognition varied depending on factors like age, dosage, treatment duration, and underlying health conditions. Preclinical and clinical studies, analyzed side by side, showed both detrimental and beneficial effects on cognition, with healthy subjects often experiencing negative impacts while those with pathological conditions exhibiting improvements. This evidence suggests that fluoxetine should be used with caution in prophylactic therapies.

Other types of CNS-acting drugs, such as psychostimulants, including illicit and prescription drugs such as cocaine, amphetamines, methylphenidate, and modafinil, increase alertness and arousal, causing general behavioral excitement but impacting social behaviors in animals and humans. Cid-Jofre et al. highlights that psychostimulants can significantly alter social interactions such as social play, aggression, maternal care, and pair bonding due to their effects on neural circuits involved in reward and addiction. The effects vary based on factors such as the type of drug, duration of use, and whether the consumption is clinical or recreational. Moreover, the authors describe that detrimental effects are related to non-medical uses of these drugs. Therefore, the complexity of psychostimulant effects suggests that these drugs should not be viewed as a homogeneous class due to their diverse impacts on neurotransmitter systems.

In conclusion, through multiple innovative approaches, this Research Topic advanced our understanding of the effects of medicinal drugs on cognitive functions in several neuropsychiatric disorders, with both desired and undesired consequences. The papers herein integrate findings that will ultimately advance the field’s knowledge on the cognitive consequences of therapeutic drugs and inform future rational of drug use. Also, these studies emphasize the necessity for heightened caution concerning drug use due to their potentially detrimental cognitive effects, even when used prophylactically or off-label.

